# Status of eating behaviours and food education for children aged 6–35 months in Beijing, China: a cross-sectional study

**DOI:** 10.7189/jogh.16.04219

**Published:** 2026-07-24

**Authors:** Xuening Li, Yiwen Huang, Shimiao Gong, Na Meng, Chen Zhao, Qiong Wu, Yanfeng Zhang

**Affiliations:** 1 Department of Integrated Early Childhood Development, Capital Centre for Children's Health, Capital Medical University, Capital Institute of Pediatrics, Beijing, China; 2 Child Healthcare Centre, Capital Centre for Children's Health, Capital Medical University, Capital Institute of Pediatrics, Beijing, China; 3 Beijing KidsHome Children Development Centre, Beijing, China

**Keywords:** eating behaviour, food education, infant and young children, caregivers, food neophobia, picky eating

## Abstract

**Background:**

Infancy and early childhood are critical periods for establishing lifelong eating habits. Current practices regarding children aged <3 years often prioritise nutritional intake over food education. We investigated eating behaviours and food education among children aged 6–35 months in Beijing, China.

**Methods:**

We conducted a cross-sectional survey from June to September 2024 among primary caregivers of children aged 6–35 months recruited *via* convenience sampling from 388 community health centres in Beijing. We collected data *via* a self-administered online questionnaire, which participating caregivers completed by scanning the questionnaire’s quick response code on WeChat.

**Results:**

We surveyed a total of 876 caregivers. Caregivers reported picky eating in 12.7%, prolonged mealtime in 19.5%, and inattentive eating in 16.0% of children. Consumption of unhealthy foods and sugar-sweetened beverages was reported in 38.9% and 20.8% of children. A high proportion of children were classified as having moderate or severe levels of food neophobia (97.0%). Multivariable analysis identified child age, nursery attendance, and caregiver education as key factors associated with eating behaviours and dietary consumption. Although >95.0% of caregivers recognised the importance of food education, only 32.2% frequently conducted food education activities at home.

**Conclusions:**

Eating behaviour problems and food neophobia are widespread among children aged 6–35 months in Beijing. Developmentally appropriate food education may help address the awareness-practice gap and promote healthy eating habits.

Infancy and early childhood are critical periods for establishing eating behaviours, many of which track into adulthood and influence long-term health [1]. During this period, early eating experiences fundamentally shape children's acceptance of foods and dietary patterns [2]. In particular, the complementary feeding period (6–23 months of age) marks a critical transition from a milk-based diet to diversified foods, representing a sensitive window for establishing lifelong eating habits [3,4].

Food education, broadly defined as educational activities that promote knowledge about food, positive eating experiences, and engagement with food-related practices, was first proposed by Japanese researcher Sagen Ishizuka in 1861 and has evolved globally over the decades [5]. In early childhood, food education may include helping children recognise foods, encouraging positive mealtime behaviours, supporting sensory exploration, and fostering familiarity with food preparation and dietary culture. Evidence suggests that appropriate food education during complementary feeding may broaden food acceptance, reduce food neophobia, stimulate taste development, and support the development of oral-motor skills such as chewing and swallowing, which together contribute to the development of healthy eating [6]. Beyond individual health, early food education also facilitates the transmission of dietary culture and social norms. Recognising its public health importance, China’s National Health Commission issued the ‘Feeding and Nutrition Guidelines for Infants and Young Children in Nursery Institutions’ in 2021, which defined the scope of food education for infants and young children into three categories of food cognition, eating behaviours, and dietary culture, and emphasised collaboration between nursery institutions and families [7].

Despite policy guidance, feeding practices in both Chinese families and nursery institutions often prioritise dietary intake over food education, which may be associated with problematic eating behaviours. At home, caregivers may focus primarily on ensuring adequate food consumption while neglecting sensory exploration, self-feeding skills, and positive mealtimes [8]. In nursery institutions, excessive emphasis on eating speed and discipline may also be associated with coercive or distracted feeding practices, which have been suggested to be related to picky eating, food selectivity, and delayed oral motor development [9]. Currently, research on food education in China has predominantly focused on older children, with limited evidence available for those aged <3 years, a population highly dependent on family and nursery environments.

To address this gap, we investigated eating behaviours and food education status among children aged 6–35 months in Beijing, aiming to inform targeted feeding strategies and improve food education systems. In this study, food education practices refer to caregiver-reported activities aiming to help young children recognise foods, develop positive mealtime behaviours, and learn about dietary culture.

## METHODS

### Study design and participants

We conducted a cross-sectional survey in Beijing, China, between June and September 2024. Participants were primary caregivers of children aged 6–35 months in 388 community health centres across 15 districts of Beijing. The inclusion criteria were: children aged 6–35 months; caregivers who could read Mandarin, used WeChat (Tencent Holdings Ltd, Shenzhen, China), and had internet access. The exclusion criteria were: children with structural or genetic birth defects, including neural tube defects, congenital heart disease, and phenylketonuria; and caregivers who refused to participate.

### Sample size and sampling

We calculated the sample size for estimating a single population proportion using the formula n = Z^2^p(1 – p)/d^2^ where ‘n’ is the required sample size, ‘Z’ is the standard normal variate corresponding to the 95% confidence level (1.96), ‘p’ is the expected prevalence, and ‘d’ is the absolute precision. Assuming a prevalence of household food education implementation of 50% (*P* = 0.50) and an absolute precision of 0.055 (d = 0.055), the minimum required sample size was 318. After accounting for a 20% non-response rate, the target sample was 398 participants. A convenience sampling method was used to recruit caregivers. Due to logistical constraints, we recruited two to three eligible primary caregivers from each community health centre.

### Survey instrument

We developed the survey instrument based on a review of existing literature on food education and related eating behaviours, followed by discussions within the study team. Relevant questionnaire items from previous studies were identified and adapted to the context of caregivers of young children in Beijing. The draft questionnaire was further reviewed by 16 maternal and child health professionals from district-level maternal and child health hospitals in Beijing to improve the clarity, relevance, and comprehensibility of the questions. A pilot test involving 10 caregivers of children aged 6–35 months was conducted to assess the questionnaire’s feasibility and clarity, and minor wording revisions were made accordingly.

The final questionnaire comprised three main sections – socio-demographic characteristics of the caregivers and children, caregivers’ knowledge, attitudes, and practices regarding food education, and children’s eating behaviours (Appendix S1 in the **Online Supplementary Document**). We also included the Chinese version of the Child Food Neophobia Scale (CFNS) in the questionnaire, which consisted of six items and was scored on a seven-point Likert scale (1 = strongly disagree, 7 = strongly agree), with total CFNS scores ranging from 6–42 [10].

### Data collection

We collected the data using the online survey platform Sojump (Changsha Ranxing Information Technology Co., Ltd, Changsha, China). The survey instrument was set up on the platform, which generated a quick response (QR) code linked to the questionnaire. Health workers in each community health centre invited primary caregivers to participate in the study and guided them to access the questionnaire by scanning the QR code *via* WeChat. Caregivers then completed the online questionnaire independently.

### Outcomes

The primary outcomes were children’s eating behaviours, including picky eating, prolonged mealtime (≥30 minutes), and inattentive eating. Picky eating was defined in the questionnaire as frequently refusing certain foods or accepting only a limited variety. Inattentive eating refers to situations in which the child is easily distracted during meals, such as playing with toys or watching television. We assessed these behaviours using caregiver-reported questions about the child’s eating behaviour over the past month. Caregivers were asked how frequently their child exhibited each behaviour, with response options including never, rarely, sometimes, often, and always. We calculated the proportion of caregivers who reported that their children often or always exhibited each behaviour. Inability to eat independently was defined as situations in which the child required full assistance from caregivers during meals, based on caregivers' responses to whether the child could eat independently at home. Consumption of unhealthy foods or sugar-sweetened beverages was also assessed, defined as the proportion of children who consumed such foods in the previous 24 hours. Unhealthy foods included sweets (*e.g.* chocolate, candy, cake, biscuits, ice cream or popsicles) and puffed or fried foods (*e.g.* potato chips, French fries, shrimp crackers, fried doughnuts, choux pastry, and instant noodles). Sugar-sweetened beverages included fruit juice, cola, honey water, sugar water, and lactic acid drinks.

The secondary outcome was food neophobia, referring to a child’s reluctance to eat or avoidance of novel foods. Using the CFNS, we categorised children into three levels of food neophobia based on quartile distribution of the total scores in the study sample: mild food neophobia (scores ≤14.5), moderate food neophobia (scores between 14.5 and 33.5), and severe food neophobia (scores ≥33.5) [10]. We used these categories to describe relative levels of food neophobia within the study population. The proportion of children at a moderate or severe level of food neophobia was reported.

The third outcome was the implementation of food education and caregivers' beliefs about it. Household food education implementation refers to caregivers engaging children in food-related activities within the family setting, such as helping them recognise foods, develop positive mealtime behaviours, and learn about basic dietary culture. The proportion of food education implementation was assessed separately for households and nursery institutions defined as the percentage reporting that such activities were conducted often or always. In addition, we evaluated the approaches used by caregivers to deliver food education and their knowledge sources.

### Data management and statistical analysis

We performed all statistical analyses using SAS, version 9.4 (SAS Institute Inc., Cary, North Carolina, USA). Categorical variables were presented as numbers and frequencies (%). We tested continuous variables for normality using the Shapiro-Wilk test, and since they were non-normally distributed, we presented data as medians (Mdn) and interquartile ranges (IQR). We assessed differences in categorical variables between groups using the χ^2^ test or Fisher exact test, as appropriate. Moreover, we performed multivariable logistic regression analysis to identify factors associated with eating behaviours, including child age, caregiver type (mothers *vs.* other caregivers), caregiver education level (university degree or above *vs.* high school or below), and nursery attendance. Results were presented as odds ratios (ORs) with 95% confidence intervals (CIs). A two-sided *P*-value <0.05 was considered statistically significant.

## RESULTS

We surveyed 876 primary caregivers of children aged 6–35 months, with mothers accounting for 80.9%, followed by grandparents (11.5%) and fathers (6.7%). The vast majority of primary caregivers (90.6%) had a university degree or above (**Table 1**).

**Table 1:** Characteristics of surveyed children and primary caregivers (n = 876)

Characteristics	n (%)
**Children**	
Age in months	
*6–11*	234 (26.7)
*12–23*	351 (40.1)
*24–35*	291 (33.2)
Sex	
*Male*	467 (53.3)
*Female*	409 (46.7)
**Primary caregivers**	
Relationship with children	
*Mother*	709 (80.9)
*Father*	59 (6.7)
*Grandparents*	101 (11.5)
*Others*	7 (0.8)
Mother’s age in years, Mdn (IQR)	33 (30–36)
Educational level	
*Junior high school and below*	23 (2.6)
*High school*	59 (6.7)
*University degree and above*	794 (90.6)

IQR – interquartile range, Mdn – median

Problematic eating behaviours reported by caregivers were 12.7% for picky eating, 19.5% for prolonged mealtime (≥30 minutes), and 16.0% for inattentive eating, with the highest rates observed in children aged 24–35 months. Overall, 45.2% of children were unable to eat independently. Unhealthy food consumption and sugar-sweetened beverage consumption were reported in 38.9% and 20.8% of children. Based on the CFNS classification, 97.0% of children were categorised as having moderate or severe levels of food neophobia (**Table 2**).

**Table 2:** Eating behaviours and food education status of children at different ages (n = 876), n (%)

Items	6–11 months (n = 234)	12–23 months (n = 351)	24–35 months (n = 291)	Total (n = 876)	*P*-value
Eating behaviours					
*Picky eating*	21 (9.0)	41 (11.7)	49 (16.8)	111 (12.7)	0.0206
*Prolonged mealtime*	19 (8.1)	79 (22.5)	73 (25.1)	171 (19.5)	<0.0001
*Inattentive eating*	23 (9.8)	63 (18.0)	54 (18.6)	140 (16.0)	0.0109
*Inability to eat independently*	202 (86.3)	151 (43.0)	43 (14.8)	396 (45.2)	<0.0001
*Consumption of unhealthy foods*	26 (11.1)	134 (38.2)	181(62.2)	341 (38.9)	<0.0001
*Consumption of beverages*	18 (7.7)	68 (19.4)	96 (33.0)	182 (20.8)	<0.0001
Food neophobia					
*Mild*	12 (5.1)	9 (2.6)	5 (1.7)	26 (3.0)	0.0618
*Moderate*	194 (82.9)	303 (86.3)	267 (91.8)	764 (87.2)	0.0086
*Severe*	28 (12.0)	39 (11.1)	19 (6.5)	86 (9.8)	0.0660
General information on food education					
*Ever heard of food education*	165 (70.5)	260 (74.1)	223 (76.6)	648 (74.0)	0.2828
*Believe that children need food education*	223 (95.3)	335 (95.4)	280 (96.2)	838 (95.7)	0.8464
*Household food education implementation*	74 (31.7)	124 (35.4)	84 (28.8)	282 (32.2)	0.2133
*Children attend nursery institutions*	12 (5.1)	34 (9.7)	77 (26.5)	123 (14.0)	<0.0001
*Nursery institutions food education implementation*	4 (33.4)	18 (53.0)	42 (54.6)	64 (52.0)	0.3892
*Children attend early education centres*	15 (6.4)	36 (10.3)	60 (20.6)	111 (12.7)	<0.0001
*Early education centres often conduct food education for children*	4 (26.7)	16 (44.5)	19 (31.7)	39 (35.1)	0.3400
Caregivers’ concerns about children’s knowledge of food nutrition and safety					0.0972
*Not at all concerned*	4 (1.7)	8 (2.3)	3 (1.0)	15 (1.7)	
*Slightly concerned*	19 (8.1)	17 (4.8)	22 (7.6)	58 (6.6)	
*Moderately concerned*	63 (26.9)	119 (33.9)	113 (38.8)	295 (33.7)	
*Quite concerned*	78 (33.3)	119 (33.9)	88 (30.2)	285 (32.5)	
*Very concerned*	70 (29.9)	88 (25.1)	65 (22.3)	223 (25.5)	
Often engage the child in learning about and experiencing food	94 (40.2)	185 (52.9)	134 (46.0)	413 (47.2)	0.0108
Often involve the child in preparing food	37 (15.9)	62 (17.7)	52 (17.9)	151 (17.3)	0.7947
Often involve the child in planting, growing or harvesting activities	19 (8.1)	45 (12.8)	33 (11.4)	97 (11.1)	0.2037
Often explain food and nutrition knowledge to the child	55 (23.5)	100 (28.5)	62 (21.3)	217 (24.8)	0.0962
Attitudes towards disliked foods of the child					
*Avoid preparing the disliked food, and believe the child will outgrow the aversion*	32 (13.7)	66 (18.8)	56 (19.2)	154 (17.6)	0.1844
*Modify cooking methods to encourage the child to try disliked foods*	172 (73.5)	251 (71.5)	221 (76.0)	644 (73.5)	0.4477
*Encourage the child to eat*	137 (58.6)	218 (62.1)	181(62.2)	536(61.2)	0.6257
Experience dietary culture					
*Often explain food-related knowledge to the child during festivals*	65 (27.8)	114 (32.4)	92(31.6)	271(30.9)	0.4616
*Frequently introduce local cuisine and food culture to the child*	53 (22.7)	82 (23.3)	51(17.6)	186(21.3)	0.1634
*Frequently educate the child to appreciate food and avoid waste*	79(33.8)	148 (42.1)	136(46.8)	363(41.4)	0.0104
*Frequently instruct the child in table manners*	85 (36.3)	151 (43.1)	120(41.2)	356(40.6)	0.2627

Multivariable analysis indicated that increasing child age was independently associated with increased odds of picky eating, prolonged mealtime, inattentive eating, unhealthy food consumption, sugar-sweetened beverage consumption, and moderate or severe food neophobia (OR = 1.03–1.12), but with decreased odds of inability to eat independently (OR = 0.85; 95% CI = 0.83–0.87, *P* < 0.001) (**Table 3**). Nursery attendance was associated with reduced odds of inability to eat independently (OR = 0.52; 95% CI = 0.29–0.91, *P* = 0.0214). Caregivers with a university degree or above had decreased odds of children consuming unhealthy foods (OR = 0.46; 95% CI = 0.28–0.77, *P* = 0.0027) and sugar-sweetened beverages (OR = 0.30; 95% CI = 0.18–0.49, *P* < 0.0001). Moreover, compared with other primary caregivers, mothers were more likely to provide sugar-sweetened beverages to their children (OR = 1.66; 95% CI = 1.03–2.67, *P* = 0.0387).

**Table 3:** Multivariable analysis of eating behaviours and food consumption

Variables	Picky eating		Prolonged mealtime		Inattentive eating		inability to eat independently		Consumption of unhealthy foods		Consumption of beverages		Moderate or severe food neophobia	
	**OR (95% CI)**	***P*-value**	**OR (95% CI)**	***P*-value**	**OR (95% CI)**	***P*-value**	**OR (95% CI)**	***P*-value**	**OR (95% CI)**	***P*-value**	**OR (95% CI)**	***P*-value**	**OR (95% CI)**	***P*-value**
Children’s age in months	1.03 (1.00–1.05)	0.0296	1.04 (1.02–1.06)	0.0001	1.03 (1.00–1.05)	0.0156	0.85 (0.83–0.87)	<0.0001	1.12 (1.10–1.14)	<0.0001	1.08 (1.06–1.10)	<0.0001	1.07 (1.01–1.13)	0.0145
Caregivers	0.95 (0.57–1.57)	0.8322	1.00 (0.65–1.54)	0.9942	0.70 (0.46–1.08)	0.1059	0.68 (0.45–1.02)	0.0618	1.17 (0.80–1.71)	0.4327	1.66 (1.03–2.67)	0.0387	2.04 (0.87–4.82)	0.1025
Caregivers educational level	1.04 (0.52–2.08)	0.9219	0.93 (0.53–1.64)	0.7970	1.11 (0.58–2.12)	0.7457	1.39 (0.80–2.43)	0.2455	0.46 (0.28–0.77)	0.0027	0.30 (0.18–0.49)	<0.0001	0.87 (0.20–3.76)	0.8482
Children attend nursery institutions	0.74 (0.43–1.26)	0.2639	1.24 (0.75–2.03)	0.4069	1.25 (0.72–2.16)	0.4305	0.52 (0.29–0.91)	0.0214	1.17 (0.75–1.81)	0.4912	1.03 (0.64–1.66)	0.8967	1.20 (0.34–4.23)	0.7773

CI – confidence interval, OR – odds ratio

Over 70.0% of caregivers had ever heard of ‘food education’, and more than 95.0% believed it necessary. However, only 32.2% often conducted food education for their children at home, compared with 52.0% in nursery institutions. Regarding food perception and understanding, >90.0% of caregivers paid attention to children’s knowledge of food nutrition and safety. However, the frequency of specific educational activities varied: 47.2% involved the child in experiencing and understanding food; 24.8% often explained food-related nutrition knowledge; 17.3% often involved the child in food preparation; and 11.1% accompanied the child in planting, cultivating, or picking activities.

Caregivers adopted varied strategies when children disliked foods: 73.5% modified the cooking method, 61.2% provided guidance and encouragement, and 17.6% adopted a passive approach, believing the child would outgrow the aversion.

Regarding dietary cultural practices, 30.9% of caregivers explained dietary knowledge to their children during festivals, and 21.3% introduced local cuisine and food culture. Furthermore, 41.4% educated their children to appreciate food and avoid waste, while 40.6% regularly instructed them in table manners.

**Figure 1:**
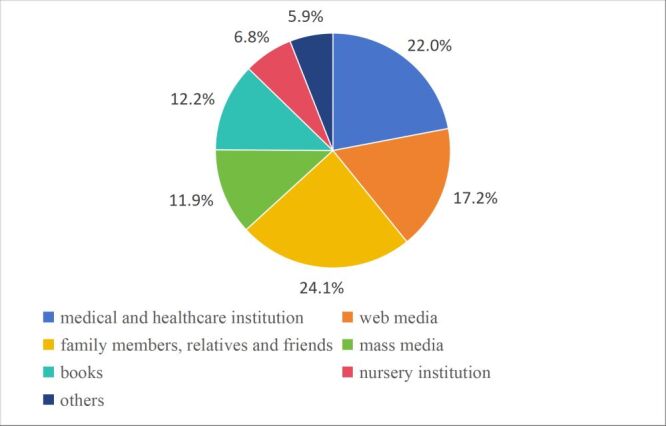
Sources of food education knowledge among caregivers.

The most common source was family members, relatives and friends, accounting for 24.1% of responses, followed by medical and health institutions (22.0%), online media (17.2%), books (12.2%), mass media (11.9%), and nursery or early education institutions (6.8%) (**Figure 1**).

**Figure 2:**
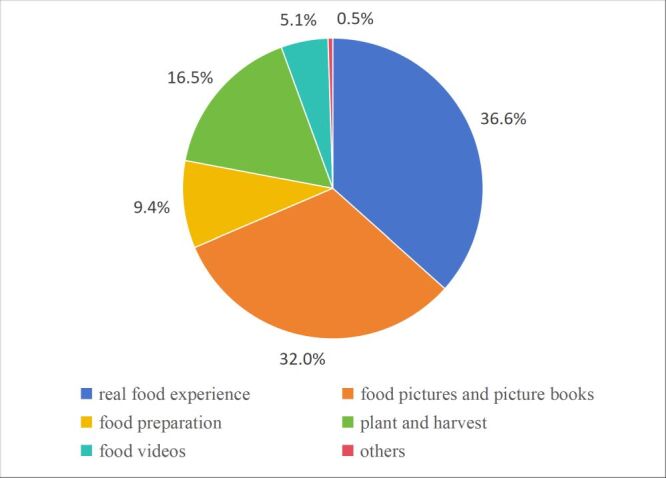
Food education approaches used by caregivers.

The most frequent method was real-food experiences, accounting for 36.6% of responses, followed by food pictures and storybooks (32.0%). Less common methods included participation in planting and picking activities (16.5%), involvement in food preparation (9.4%), and watching food-related videos (5.1%) (**Figure 2**).

## DISCUSSION

In this study, we found that eating behaviour problems, including picky eating, prolonged mealtime, inattentive eating, and consumption of unhealthy foods, are common among children aged 6–35 months in Beijing. The prevalence of picky eating (12.7%) was similar to a prior survey in Shunyi District in Beijing (13.0%) but much lower than national multi-city estimates (36.0%) [11], suggesting regional variations in feeding environments and caregiver awareness.

Notably, over 60.0% of children aged 24–35 months consumed unhealthy foods, which may impair nutrient intake and increase risks of micronutrient deficiencies and growth impairment. The age-related increase in eating behaviour problems is consistent with existing literature and likely reflects greater autonomy, broader food exposure, and a stronger influence of family dietary patterns. Early childhood, particularly between ages one and three years, constitutes a critical window for establishing lifelong dietary habits [12]. During this period of heightened neurobehavioural plasticity, inadequate guidance may contribute to readily reinforcing maladaptive feeding behaviours [13]. Therefore, early food education should emphasise positive food relationships, attentive eating, self-feeding skills, and limited intake of sugary, salty, and high-fat foods.

Age-related differences observed in this study should also be interpreted with caution. Developmental expectations for certain eating behaviours, particularly independent eating, may vary considerably across the wide age range of 6–35 months. In addition, older children are more likely to encounter a broader range of foods and eating contexts, including exposure to sugary drinks, snack foods, and more distracting mealtime environments. Therefore, the higher prevalence of certain eating behaviours and food consumption patterns among older toddlers may partly reflect increased opportunities for exposure and normal developmental progression rather than solely adverse behavioural trajectories.

The establishment of eating habits during infancy and early childhood not only results from children's individual exploration of food but also from their observation and emulation of the eating behaviours of surrounding adults [14]. This study revealed that >90.0% of children aged 6–35 months exhibit moderate to severe food neophobia, which is demonstrated as refusal to attempt new foods or avoidance of unfamiliar foods. Research suggests that food neophobia may influence children’s dietary patterns and food acceptance during early childhood [15]. Existing evidence demonstrates that food neophobia is closely associated with inadequate intake of fruits, vegetables, eggs, and fish [16,17]. Children with a high level of food neophobia are more prone to depend on familiar, unhealthy foods rich in sugar and salt, thus elevating the risk of picky eating and imbalanced nutrient intake [18,19].

Guiding young children to perceive and understand food *via* multiple sensory channels has been suggested as a potentially effective approach to stimulate their interest in food and enhance their acceptance of new foods [20]. It also constitutes the core content of food education for infants and toddlers. Relevant empirical research supports this strategy. A study conducted by the University of Reading in the UK revealed that merely through repeated visual exposure, the willingness of children aged 21–24 months to try unfamiliar fruits was significantly heightened after they viewed fruit pictures in picture books for two consecutive weeks [21]. Another study required caregivers of children aged 19–26 months to read picture books featuring unfamiliar fruits and vegetables with their children daily for two weeks. The findings indicated that this intervention effectively increased the children’s consumption of the unfamiliar vegetables and fruits depicted in the picture books [22]. Additionally, RCT had children engage in multi-sensory games involving uncommon vegetables for four weeks, and the results suggested that the children in the intervention group exhibited a greater willingness to touch and taste these vegetables [23]. These studies collectively illustrate that food education practices centred on multi-sensory interaction and enjoyable exploration may help reduce children's food neophobia and support the healthy development of their eating behaviours.

However, this study has uncovered the harsh reality of current food education practices for infants and toddlers. The survey data indicate that although >90.0% of caregivers of infants and toddlers aged 6–35 months acknowledge the importance of food education and consider it essential to provide it at home, fewer than half regularly engage in related activities. This substantial disparity between cognition and practice makes it challenging for infants and toddlers to receive effective food education support within the family, the core nurturing environment, thereby limiting the potential role of food education in supporting healthier eating behaviours. Further analysis reveals that the challenges faced by caregivers in implementing food education primarily stem from two factors. First, the acquisition of knowledge depends on non-professional sources. This study discovered that the primary sources of food education knowledge for caregivers are non-professional channels such as family, friends, and online media. The quality of information disseminated through these channels varies significantly and may even include misleading content, which impacts their understanding of scientific and appropriate food education methods. Second, there is a systematic lack of professional guidance. Currently, medical and health institutions, as well as childcare institutions, still have evident deficiencies in providing specific, operational guidance and methods for disseminating food education. Consequently, families lack clear directions and necessary support when confronted with practical issues such as ‘how to implement’ and ‘to what extent,’ making it difficult for the concept of food education to be effectively translated into continuous family practice.

Given that >80.0% of children are primarily cared for by their families, the family remains the central setting for implementing food education. The quality of information that caregivers acquire and the methodological support they can access may influence the effectiveness of food education practices. Therefore, medical and health institutions, together with nursery institutions, could play an important role in providing evidence-informed guidance and resources to support families in implementing appropriate food education practices.

However, our study has limitations. First, the convenience sampling method was used, and only two to three caregivers were recruited from each community health centre. This approach may introduce selection bias and limit the sample’s representativeness, thereby affecting the generalisability of the findings. In addition, participants were required to read Mandarin and use WeChat with internet access to complete the survey, which may have favoured more educated and digitally connected caregivers and potentially underrepresented more vulnerable families. Second, although the questionnaire items were adapted from previous studies and reviewed by professionals, formal psychometric validation, such as assessment of construct validity, test-retest reliability, or internal consistency, was not conducted. Future studies are needed to further evaluate the reliability and validity of the newly developed items. Third, caregiver self-reported data are susceptible to recall and social desirability biases, which could affect accuracy. Moreover, the CFNS has been validated mainly among children aged 12–36 months, and its applicability to younger infants aged 6–11 months is uncertain. As many infants in this age group are just beginning complementary feeding, the high prevalence observed may partly reflect developmental factors rather than true problematic food neophobia. Finally, this study used a cross-sectional design, which limits the ability to establish causal relationships or determine the directionality between food education practices and children’s eating behaviours. Longitudinal studies are needed to further clarify these relationships.

## CONCLUSIONS

Eating behaviour problems and food neophobia are widespread among children aged 6–35 months in Beijing, especially older toddlers. Developmentally appropriate food education, stressing repeated, multisensory food exposure combined with strengthened professional support, may help address the awareness-practice gap and promote healthier dietary habits.

## Additional material


Online Supplementary Document

## Data Availability

**Data availability:** The data that support the findings of this study are not publicly available due to privacy restrictions, but are available from the corresponding author upon reasonable request (summyzh@126.com).

## References

[1] World Health Organization. Essential nutrition actions: mainstreaming nutrition through the life-course. 2019. Available: https://www.who.int/publications/i/item/9789241515856. Accessed: 10 August 2025.

[2] Nicklaus S The role of food experiences during early childhood in food pleasure learning. Appetite. 2016;104:3–9. 10.1016/j.appet.2015.08.022 26298009

[3] Lutter CK Grummer-Strawn L Rogers L Complementary feeding of infants and young children 6 to 23 months of age. Nutr Rev. 2021;79:825–46. 10.1093/nutrit/nuaa143 33684940

[4] Ventura AK Worobey J Early influences on the development of food preferences. Curr Biol. 2013;23:R401–8. 10.1016/j.cub.2013.02.037 23660363

[5] Sagen I. The theory of chemical feeding and longevity. Tokyo, Japan: Bowen Hall; 1896. Japanese.

[6] Birch LL Doub AE Learning to eat: birth to age 2 y. Am J Clin Nutr. 2014;99:723S–8S. 10.3945/ajcn.113.069047 24452235

[7] Chinese Center for Disease Prevention and Control. Feeding guidelines for infants. 2022. Available: https://en.chinacdc.cn/health_topics/nutrition_health/202203/t20220301_257287.html. Accessed: 10 August 2025.

[8] Wen YJ Long C Cui HL Fang YJ Wang K Shan XY A video observation study to explore the current situation of caregivers’ pressure-to-eat feeding practices on infants and toddlers. Chin J Women Child Health. 2024;15:30–8.

[9] Wu Q Gong S Li X Zhao C Li L Huang Y Food education for infants and young children aged 0-3 years in China. China CDC Wkly. 2025;7:672–4. 10.46234/ccdcw2025.109 40376179 PMC12075495

[10] Zou J Liu Y Yang Q Liu H Luo J Ouyang Y Cross-cultural adaption and validation of the Chinese version of the child food neophobia scale. BMJ Open. 2019;9:e026729. 10.1136/bmjopen-2018-026729 31439595 PMC6707651

[11] Kwon KM Shim JE Kang M Paik HY Association between Picky Eating Behaviors and Nutritional Status in Early Childhood: Performance of a Picky Eating Behavior Questionnaire. Nutrients. 2017;9:463. 10.3390/nu9050463 28481251 PMC5452193

[12] Kawecka P Kostecka M The role of the family environment and parental nutritional knowledge in the prevention of behavioral feeding disorders in toddlers and preschool children – a narrative review. J Health Inequal. 2024;10:56–63. 10.5114/jhi.2024.140767

[13] Catena-Verdejo E Nieto-Ruiz A García-Santos JA Herrmann F De-Castellar R Pérez-Hernández MT Understanding the developmental trajectory of behavioral problems and subcortical structure morphometry in healthy children at 6 years old and long-term impact of early nutrition: the COGNIS study. Child Adolesc Psychiatry Ment Health. 2026;20:43. 10.1186/s13034-026-01030-7 41715152 PMC13020149

[14] Wang X Wu L Liu Q Wu Y Dietary environment in early care and education settings and young children’s eating behavior: a systematic review of literature. Am J Health Behav. 2022;46:541–57. 10.5993/AJHB.46.5.5 36333831

[15] Dovey TM Staples PA Gibson EL Halford JC Food neophobia and ‘picky/fussy’ eating in children: a review. Appetite. 2008;50:181–93. 10.1016/j.appet.2007.09.009 17997196

[16] Perry RA Mallan KM Koo J Mauch CE Daniels LA Magarey AM Food neophobia and its association with diet quality and weight in children aged 24 months: a cross sectional study. Int J Behav Nutr Phys Act. 2015;12:13. 10.1186/s12966-015-0184-6 25889280 PMC4335451

[17] Dovey TM Staples PA Gibson EL Halford JCG Food neophobia and “picky/fussy” eating in children: a review. Appetite. 2008;50:181–93. 10.1016/j.appet.2007.09.009 17997196

[18] Torres TO Gomes DR Mattos MP Factors associated with food neophobia in children: systematic review. Rev Paul Pediatr. 2020;39:e2020089. 10.1590/1984-0462/2021/39/2020089 33175005 PMC7649857

[19] Fu X Tang MY Liu XM Research progress in food neophobia in children. Chin J Child Health Care. 2024;32:68–72.

[20] Del Campo C Bouzas C Tur JA Risk factors and consequences of food neophobia and pickiness in children and adolescents: a systematic review. Foods. 2024;14:69. 10.3390/foods14010069 39796359 PMC11720204

[21] Houston-Price C Butler L Shiba P Visual exposure impacts on toddlers’ willingness to taste fruits and vegetables. Appetite. 2009;53:450–3. 10.1016/j.appet.2009.08.012 19744533

[22] Heath P Houston-Price C Kennedy O Let’s look at leeks! Picture books increase toddlers’ willingness to look at, taste and consume unfamiliar vegetables. Front Psychol. 2014;5:191. 10.3389/fpsyg.2014.00191 24653709 PMC3949128

[23] Coulthard H Sealy A Play with your food! Sensory play is associated with tasting of fruits and vegetables in preschool children. Appetite. 2017;113:84–90. 10.1016/j.appet.2017.02.003 28202412

