# Supplementary material for: Status of eating behaviours and food education for children aged 6–35 months in Beijing, China: a cross-sectional study
Source: J Glob Health. 2026 Jul 24;16:04219. doi: 10.7189/jogh.16.04219 (PMC13397868; doi:10.7189/jogh.16.04219)
Supplement: Online Supplementary Document [file jogh-16-04219-s001.pdf]

Supplement to: Li X, Huang Y, Gong S, Meng N, Zhao C, Wu Q, Zhang Y. Status of eating behaviours and food education for children aged 6–35 months in Beijing, China: a cross-sectional study. J Glob Health. 2026;16:04219.

Infant and Young Child Food Education Questionnaire

Basic Information

1. Child's name:__________________________

2. Child's gender: ○ Male ○ Female

3. Child's date of birth: _______ year _______ month _______ day

4. Child's primary caregiver: ○ Mother ○ Father ○ Grandparents ○ Maternal grandparents ○ Nanny ○ Other

5. Primary caregiver's education level: ○ Primary school or below ○ Junior high school ○ Senior high school ○ University ○ Postgraduate or above

6. Primary caregiver's age (years): _________

7. Mother's name: _________

8. Mother's education level: ○ Primary school or below ○ Junior high school ○ Senior high school ○ University ○ Postgraduate or above

9. Mother's age: _________

10. Place of residence: _______ city _______ district

Food Education

1. Have you heard of "food education"?
   ○ Yes ○ No (skip to question 3)

2. Where did you hear about "food education"?
   ○ Relatives or friends
   ○ Nursery or early education institution teachers
   ○ Hospital or community health center doctors
   ○ Books
   ○ Mass media (radio, TV, newspapers, etc.)
   ○ Online media (websites, WeChat, TikTok, Xiaohongshu, etc.)
   ○ Other _______

3. Do you think infants need food education?

○ Yes ○ No

4. What do you think infant’s food education should include?

○ Knowing and experiencing food (food names, categories, colors, shapes, sizes, smells, tastes, sources, production and processing, etc.)

○ Nutrition and health knowledge

○ Cultivation of good eating behavior habits

○ Food culture

○ Cherishing food

○ Environmental awareness

○ Other _______

5. Do you think food education for infants should be carried out at home?

○ Yes ○ No

6. Have you ever carried out food education with your child at home?

○ Never ○ Rarely ○ Sometimes ○ Often ○ Always

7. On average, how much time do you spend on food education with your child at home per day?

○ Never do food education ○ Less than 10 minutes ○ 10–20 minutes ○ More than 30 minutes

8. In which situations do you usually carry out food education with your child?

○ When cooking, tell the child what the food is

○ When eating, tell the child what the food is

○ During traditional festivals

○ When going fruit/vegetable picking

○ When shopping at supermarkets or wet markets

○ When the child shows poor eating behaviors or habits

○ Doctor's advice

○ Teacher's advice

○ Advice from relatives or friends

○ Never do it

○ Other _______

9. Where do you obtain knowledge about food education to teach your child?

○ Family members

○ Friends

○ Other parents

○ Nursery or early education institution teachers

○ Hospital or community health center doctors

○ Books

○ Mass media (radio, TV, newspapers, etc.)

○ Online media (websites, WeChat, TikTok, Xiaohongshu, etc.)
○ Other _______

10. Does your child attend a nursery?
○ Yes ○ No

11. Does your child attend an early education institution?
○ Yes ○ No

12. Do you wish nursery/early education institutions to carry out food education activities?
○ Yes ○ No

13. Does the nursery or early education institution your child attends carry out food education with the child?
○ Never ○ Rarely ○ Sometimes ○ Often ○ Always

Experiencing and Knowing Food

1. How much attention do you pay to knowledge about child food nutrition and safety?
○ Never pay attention: do not actively seek or refuse to learn food-related knowledge
○ Pay little attention: only seek and learn relevant knowledge when solving child's eating problems
○ Average attention: neither active nor resistant; learn when relevant knowledge appears around
○ Relatively attentive: rarely actively seek some food knowledge
○ Very attentive: actively and often seek and learn child food-related knowledge

2. Do you take your child to know and experience food at home?
○ Never ○ Rarely ○ Sometimes ○ Often ○ Always

3. How do you help your child experience and know food?
○ Food pictures or cards
○ Experience real food with five senses (touch, smell, taste, listen, look)
○ Picture books
○ Take the child to participate in planting
○ Take the child fruit/vegetable picking
○ Let the child participate in food preparation and making (e.g., vegetable picking, washing, kneading dough, stirring)
○ Play food-related games
○ Play food-related videos
○ Show the child while cooking or eating
○ Have never done it
○ Other _______

4. Do you create opportunities for parent-child food making?

○ Never ○ Rarely ○ Sometimes ○ Often ○ Always

5. Have you accompanied your child in planting, growing, or picking activities?
   ○ Never ○ Rarely ○ Sometimes ○ Often ○ Always

6. Do you and your family explain food nutrition knowledge to your child?
   ○ Never ○ Rarely ○ Sometimes ○ Often ○ Always

7. Please rate your child's mastery of the following knowledge:

- Specific names of foods: ○completely unaware ○not very aware○ basically aware○ relatively aware○ very aware
- Characteristics of foods (shape, taste, etc.): ○completely unaware ○not very aware○ basically aware○ relatively aware○ very aware
- Colors of foods: ○completely unaware ○not very aware○ basically aware○ relatively aware○ very aware
- Source and growth process of foods: ○completely unaware ○not very aware○ basically aware○ relatively aware○ very aware
- Types of foods: ○completely unaware ○not very aware○ basically aware○ relatively aware○ very aware
- Nutrients in foods: ○completely unaware ○not very aware○ basically aware○ relatively aware○ very aware

8. Based on your recollection of your child's behavior when trying new foods in the past three months, please indicate whether you agree with the following statements.

1 strongly disagree 2 very disagree 3 disagree 4 somewhat agree 5 agree 6 very agree 7 strongly agree

- My child constantly tries new and different foods.
- My child is distrustful / uneasy about new foods.
- If my child does not know what is in a food, he/she will not try it.
- My child is afraid to eat foods he/she has never eaten before.
- My child is picky about the foods he/she eats.
- My child eats almost everything.

Cultivating Eating Behaviors

1. Did your child eat any of the following foods yesterday? (Include at home and outside, from 6 a.m. yesterday to 6 a.m. today)

- Did your child eat yesterday: grains (thick porridge, rice, noodles, steamed buns, bread, biscuits, corn, etc., excluding thin porridge), white-fleshed tubers (potatoes, cassava, yams, etc.)
  ○ Yes ○ No

- Did your child eat yesterday: orange-fleshed tubers (sweet potatoes, etc.), dark green leafy vegetables (spinach, lettuce, rape, bok choy, lettuce, etc.), yellow or orange vegetables or fruits (pumpkin, carrot, tomato, persimmon, mango, orange, orange, etc.)

○ Yes ○ No

- Did your child eat yesterday: other fresh vegetables and fruits (e.g., apple, banana, pear, grape, Chinese cabbage, cauliflower)

○ Yes ○ No

- Did your child eat yesterday: meat (beef, pork, lamb, chicken, duck, etc.), fish, shrimp or other seafood, animal organs (liver, kidney, heart, etc.) and animal blood (chicken blood, duck blood, pig blood, etc.)

○ Yes ○ No

- Did your child eat yesterday: eggs (chicken, duck, goose, quail eggs, etc.)

○ Yes ○ No

- Did your child eat yesterday: beans and bean products (tofu, soymilk, dried tofu, bean curd sheet, etc.), nuts (walnuts, cashews, almonds, melon seeds, peanuts, etc.)

○ Yes ○ No

- Did your child eat yesterday: dairy products (cheese, thick yogurt, etc.)

○ Yes ○ No

- Did your child eat yesterday: additional iron supplements, such as iron tablets, iron candies, nutrient packets, etc.

○ Yes ○ No

- Did your child eat yesterday: sweets (e.g., chocolate, candy, cake, biscuits, ice cream or popsicles) and puffed or fried foods (e.g., potato chips, French fries, shrimp crackers, fried doughnuts, choux pastry, and instant noodles)

○ Yes ○ No

- Did your child drink yesterday: any sugary drinks (e.g., fruit juice, cola, honey water, sugar water, and lactic acid drinks)

○ Yes ○ No

- Did your child drink yesterday: milk

○ Yes ○ No

2. Did your child drink infant formula or cow's milk, goat's milk yesterday? (from 6 a.m. yesterday to 6 a.m. today)

○ Yes ○ No

2.1 How many times did your child drink infant formula yesterday? _______ times

2.2 How many times did your child drink cow's milk or goat's milk yesterday? _______ times

2.3 How many times did your child drink yogurt yesterday? _______ times

3. How many main meals or snacks did your child eat yesterday? Include those eaten with adults (from 6am yesterday to 6am today). Examples: rice cereal, thick porridge, noodles, steamed buns, biscuits, bread, meat, fish, fruit, vegetables, eggs, tofu, nuts, etc.; exclude water, thin porridge, rice water, drinks, etc.; a small amount of snack (just one or two bites) does not count.

○ 0 times ○ 1 time ○ 2 times ○ 3 times ○ 4 times ○ 5 times or more

4. Can your child eat independently at home? ○ Yes ○ No

5. Usually, how long does your child take to eat a meal at home?

○ Within 30 minutes ○ 30–60 minutes ○ More than 60 minutes

6. What is your child's eating state at home?

○ Child eats independently, relatively stable mood
○ Child needs to be fed by an adult, relatively stable mood
○ Child likes to play while eating, unstable mood
○ Child often has anorexia, unstable mood

7. Please rate your child's preference for the following foods:

- Grains and tubers (rice, noodles, potatoes, sweet potatoes, etc.): ○ hate ○ dislike ○ average ○ like ○ like very much ○ not applicable
- Vegetables and fungi (green vegetables, radishes, mushrooms, etc.):○ hate ○ dislike ○ average ○ like ○ like very much ○ not applicable
- Fruits (apple, banana, pear, etc.): ○ hate ○ dislike ○ average ○ like ○ like very much ○ not applicable
- Legumes (soybeans, tofu, bean products, etc.): ○ hate ○ dislike ○ average ○ like ○ like very much ○ not applicable
- Eggs (chicken, duck, goose eggs): ○ hate ○ dislike ○ average ○ like ○ like very much ○ not applicable
- Dairy (milk, yogurt, cheese, etc.): ○ hate ○ dislike ○ average ○ like ○ like very much ○ not applicable
- Meat, poultry, fish (seafood, chicken, duck, beef, lamb, etc.): ○ hate ○ dislike ○ average ○ like ○ like very much ○ not applicable
- Sweets and sugars (ice cream, cake, candy, etc.): ○ hate ○ dislike ○ average ○ like ○ like very much ○ not applicable
- High-fat fast food (burger, fried chicken, fries, etc.):○ hate ○ dislike ○ average ○ like ○ like very much ○ not applicable
- Snacks and drinks (potato chips, biscuits, milk tea, etc.): ○ hate ○ dislike ○ average ○ like ○ like very much ○ not applicable

8. What attitude and approach do you and your family take toward foods your child dislikes?

○ If the child doesn't like it, try not to cook it, cook what the child likes; they will grow out of it
○ Try to change the cooking method to let the child try the disliked food
○ Guide and encourage the child to eat it

○ Other _______

9. Based on your child's actual situation at home this month, choose the answer that best fits:
- Picky eating:
  ○ Never ○ Rarely ○ Sometimes ○ Often ○ Always
- Anorexia (loss of appetite / refusal to eat):
  ○ Never ○ Rarely ○ Sometimes ○ Often ○ Always
- Eating too fast (less than 10 minutes):
  ○ Never ○ Rarely ○ Sometimes ○ Often ○ Always
- Eating too slowly (more than 30 minutes):
  ○ Never ○ Rarely ○ Sometimes ○ Often ○ Always
- Not eating on time (irregular meals):
  ○ Never ○ Rarely ○ Sometimes ○ Often ○ Always
- Distracted eating (talking, playing with toys, watching TV, etc.):
  ○ Never ○ Rarely ○ Sometimes ○ Often ○ Always
- Always eating unhealthy snacks (fried/puffed, high-sugar desserts, sugary drinks, etc.):
  ○ Never ○ Rarely ○ Sometimes ○ Often ○ Always
- Likes to eat fruits and vegetables, etc.:
  ○ Never ○ Rarely ○ Sometimes ○ Often ○ Always

10. What food-related work does your child's nursery do? (multiple choices)
○ Not attending nursery
○ Weekly recipe sharing
○ Communicate with parents about children's eating at the nursery
○ Share food education knowledge with parents
○ Invite parents to participate in children's meal times
○ Invite parents to participate in children's food education lessons
○ Invite parents to participate in children's planting and food making activities
○ Other _______

Experiencing Food Culture

1. What table manners do you know?
○ Wash hands before and after meals
○ Wear a bib
○ Elders sit first, younger ones sit later
○ Wait until everyone is seated and elders start eating before eating
○ Eat without making loud noises
○ Do not "rummage" through dishes when picking food
○ Take only as much as you will eat
○ Do not "force" to pick dishes that are out of reach
○ Do not do things unrelated to eating while eating
○ Do not leave the table before the meal ends
○ Other _______

2. How well does your child understand traditional festival foods?

○ Can match a few traditional festivals with their foods

○ Can match most traditional festivals with their foods

○ Can introduce traditional festivals and their corresponding foods to others

○ Child does not have a clear concept of traditional festivals

3. During festivals, do you tell your child why we eat certain festive foods (e.g., zongzi, mooncakes)?

○ Never ○ Rarely ○ Sometimes ○ Often ○ Always

4. Do you introduce local delicacies and food culture to your child?

○ Never ○ Rarely ○ Sometimes ○ Often ○ Always

5. Do you teach your child that food comes from labor and we should be grateful and not waste it?

○ Never ○ Rarely ○ Sometimes ○ Often ○ Always

6. Do you teach your child table manners?

○ Never ○ Rarely ○ Sometimes ○ Often ○ Always

7. Does your child help with pre-meal preparation and post-meal cleanup?

○ Child is too young to help

○ Yes, and is very willing to do it

○ Child likes to do it, but I / the grandparents do not allow it

○ Rarely ask the child, but the child can do it
